# 892. Misuse of Amnion and Cellular Tissue Products: A Review of Medicare Fraud and Regulatory Responses

**DOI:** 10.1093/jbcr/irag033.378

**Published:** 2026-04-06

**Authors:** Steven A Kahn, Taryn E Travis, James H Holmes, IV, Jeffrey E Carter

**Affiliations:** Medical University of South Carolina, Charleston, SC; The Burn Center at MedStar Washington Hospital Center, Washington, DC; Atrium Health Wake Forest Baptist Burn Center, Winston-Salem, NC; University Medical Center Burn Center, Louisiana State University Health Sciences Center, New Orleans, LA

## Abstract

**Introduction:**

The recent explosion of “advanced” skin substitutes and wound care products, particularly amnion-based (Am) and cellular tissue products (CTPs), has catalyzed exponential escalation in Medicare spending. This paradigm has exposed wide insurance vulnerabilities, exploited by systemic health care fraud schemes. The purpose of this review is to highlight increasing expenditures along with fraud and subsequent enforcement/regulatory responses to highlight the critical need for evidence-based practice and responsible Am/CTP utilization.

**Methods:**

A systematic review of fraud regarding Am/CTPs was conducted, including official documents released between 2024-2025 by the U.S. DOJ, HHS-OIG, and CMS. Key data reviewed included the 2025 National Health Care Fraud Takedown results, findings from the September 2025 HHS-OIG report on Am/CTP spending, finalized/proposed CMS payment and coverage rules for 2026, and lay-press publications about these topics. Increases in Am/CTP expenditures, common fraud paradigms, and enforcement actions were classified and summarized in discrete patterns.

**Results:**

Nineteen stories and official documents were reviewed, with approximately $1.2 billion in fraudulent claims for medically unnecessary CTPs, targeting vulnerable and terminally ill Medicare beneficiaries. Perpetrators were found to have targeted vulnerable populations in home-based settings, received illegal kickbacks, used medically untrained sales teams, utilized oversized grafts, upcoding, product switching, submitting multiple claims per day, insufficient documentation, and lack of medical necessity (i.e., putting CTP on a scrape) for maximum reimbursement. Most notably, high-volume claims were submitted from unrelated specialties such as psychiatry. Medicare Part B spending on CTPs ballooned from $256 million in 2019 to $10 billion in 2024. In response, the DOJ's 2025 Takedown charged perpetrators for over $1B in fraudulent Am/CTP claims, with lead parties pleading guilty with major penalties. Effective 1/1/26, CMS Local Coverage Determinations will restrict CTP use by requiring a minimum 4 weeks of “failed standard care,” limiting number of Am/CTP applications, and defining covered products. CMS also proposed a 2026 standard payment rate to eliminate the incentive for spread pricing.

**Conclusions:**

Over $1B in fraud shrouding Am/CTPs demonstrates that unchecked monetary incentives can severely impair patient care and system integrity. The upcoming regulatory changes emphasize that responsible, evidence-based use of Am/CTPs is paramount.

**Applicability of Research to Practice:**

Providers must align clinical practice and documentation with the 2026 standards to ensure appropriate patient access while safeguarding financial responsibility.

**Funding for the study:**

N/A.

